# ‘*Weak by Structure*’—Limb Muscle Fibre Cytoarchitecture Remodelling During Critical Illness and Effects of Chaperone Co-Inducer BGP-15 and Dissociative Glucocorticoid VBP-15

**DOI:** 10.3390/cells15131219

**Published:** 2026-07-04

**Authors:** Julian Bauer, Sofia Mnuskina, Anette Wirth-Hücking, Michael Haug, Dominik Schneidereit, Stefanie Nübler, Lucas Kreiß Roohian, Sebastian Schürmann, Nicola Cacciani, Lars Larsson, Oliver Friedrich

**Affiliations:** 1 Institute of Medical Biotechnology, Department of Chemical and Biological Engineering, Friedrich-Alexander University Erlangen-Nürnberg, Paul-Gordan-Str. 3, 91052 Erlangen, Germany; julian.bauer@fau.de (J.B.); anette.wirth-huecking@fau.de (A.W.-H.); michael.haug@fau.de (M.H.); stefanie.nuebler@fau.de (S.N.); lucas.kreiss@fau.de (L.K.R.); sebastian.schuermann@fau.de (S.S.); 2 Erlangen Graduate School in Advanced Optical Technologies (SAOT), Friedrich-Alexander-Universität Erlangen-Nürnberg, 91052 Erlangen, Germany; 3 Department of Clinical Sciences, Swedish University of Agricultural Sciences, SE-750 07 Uppsala, Sweden; nicola.cacciani@slu.se

**Keywords:** critical illness myopathy, edl muscle, *soleus* muscle, Second Harmonic Generation, BGP-15, quantitative morphometry, 3D cytoarchitecture, remodelling

## Abstract

Critical illness myopathy (CIM) is linked to mechanical ventilation and complete mechanical muscle silencing in intensive care unit (ICU) patients. Limb muscles show atrophy and declined specific single fibre force through altered protein turnover and diminished myosin-to-actin ratios. A rat ICU model reproducing preferential myosin loss and specific force decline in limb muscle was used to assess myofibrillar remodelling. After 5 or 10 days of ICU intervention, single *extensor digitorum longus* (EDL) and *soleus* muscle fibres were imaged using label-free, *Second Harmonic Generation* (SHG) microscopy, followed by quantitative 3D morphometry. The degree and severity of deranged myofibrillar architecture was assessed through (i) 2D and 3D *Cosine Angle Sums* (CAS2D/CAS3D) and (ii), *Vernier Densities* (VD) parameters. A progressively declining myofibrillar order was seen by dropping CAS2D/3D and increasing VD values during ICU intervention, reflecting angular and axial register deviations. Effects of chaperone co-inducer BGP-15, dissociative glucocorticoid Vamorolone (VBP-15) or its parent compound Prednisolone on myofibrillar architecture were explored. Both BGP-15 and VBP-15 modulated the progression of myofibrillar disorder seen during ICU intervention alone: For *soleus* fibres, BGP-15 maintained structural integrity at day 5 but not at day 10, while even worsening myofibrillar order in the EDL. VBP-15 reversed atrophy at day 10 in *soleus* but not in EDL fibres. Our study is the first to quantify myofibrillar remodelling in limb muscle fibres during ICU intervention in 3D and provides exploratory assessment of BGP-15 and VBP-15 treatments on aberrant remodelling in CIM.

## 1. Introduction

Critical illness myopathies (CIM) or intensive care unit (ICU)-acquired myopathies represent one of the most common neuromuscular sequelae of critical illness [1,2,3]. They are characterised by limb and trunk muscle weakness that goes beyond the effects of pure atrophy, as marked by a preferential myosin loss [1,4,5]. This renders patients bedridden for longer periods of time in the ICU and ventilator-dependent as the diaphragm is also affected [6,7,8]. For a long time, the confounding variables responsible for CIM were suspected to be the triad of neuromuscular blockade (NMBA), steroids and sepsis, as all of those interfere with protein turnover in muscle [9,10,11] and are usually all present in combination in most ICU patients, as found in multi-centre clinical studies [4]. Unlike the condition in the ICU-treated patient who is usually intubated and mechanically ventilated under neuromuscular blockade (also leading to complete mechanical silencing), septic and/or under the treatment of corticosteroids, animal models allow the separation and titration of those contributors. However, care has to be taken, as some models do replicate muscular weakness but not the human pathology of CIM. In particular, the major hallmark of CIM is seen in a preferential myosin loss with decreased myosin:actin protein ratios and diminished specific force (force per cross-sectional area) [12,13,14]. However, this is not reproduced by sepsis alone in most rodent models where single fibre atrophy and decline in absolute force prevails but specific force may be either unchanged (i.e., compatible with a pure atrophy-related weakness [15], or reduced [16]. This all depends on the model [17] and even muscle type, i.e., fast- vs. slow-twitch [18,19].

With the implementation of elaborate animal models mimicking the human ICU condition of mechanical ventilation (MV) and NMBA, all hallmarks of CIM could be reproduced both in a rat [5,20] and a porcine ICU model [21,22,23]. Those included preferential loss of myosin, transcriptional down-regulation of myosin synthesis, muscle atrophy and a marked decrease in single muscle fibre specific force generation, both in *extensor digitorum longus* (EDL) and *soleus* muscle [5]. In using those models, the MV+NMBA condition represents the minimal requirement mimicking the human ICU-patient, but unlike in the latter, risk factors can be added in isolation or combined (e.g., MV/NMBA/steroids/sepsis) [23]. From those animal models, it became clear that complete mechanical muscle silencing was the primary factor to drive the CIM phenotype [24]. This was also exacerbated by the mechanical stress on the lungs exerted through external ventilation. While mechanical silencing drives the preferential myosin loss seen in CIM, MV further compromises specific force secondary to post-translational modifications (PTMs) of myosin also in the absence of preferential myosin loss [6].

So far, compromised ‘quality’ of muscle contraction in CIM has involved contributions from changes to muscle regulatory proteins (i.e., PTMs), altered protein expression and turnover as well as altered Ca^2+^ homeostasis during contraction [25]. While all those regulate force output on a short-to-medium time-scale, the preferential myosin loss together with less orderly coordinated motor protein synthesis and filament anchorage may result in aberrant sterical arrangement of the myofibrillar lattice. Therefore, a marked deviation from the normally perfectly aligned sarcomere structure could represent a structural long-term fixation of motor protein turnover in CIM [1]. Electron microscopy has confirmed preferential myosin loss as thick filament thinning within sarcomeres with high spatial resolution [26]; however, this imaging modality precludes a detailed analysis of the three-dimensional sarcomere architecture. This is where label-free *Second Harmonic Generation* (SHG) multiphoton imaging has proven invaluable in mapping the sterical architecture of sarcomeres within muscle fibres with micron resolution to quantify ultrastructural remodelling in health, disease and ageing [27,28,29,30]. Using quantitative morphometry, myofibrillar angular orientations as well as axial lattice disruptions can be extracted and reliably described by two single parameters: *Cosine Angle Sums* (CAS) and *Vernier Densities* (VD) [28,31,32]. These two parameters represent descriptors of myofibrillar angular and axial lattice order. Specifically, CAS is close to unity and VD has low values in perfectly aligned myofibrils in healthy muscle fibres, but they inversely change with degree of disorder (i.e., CAS dropping and VD increasing) [29,31]. We recently applied this unique approach to the rat ICU model [5] and found that sarcomere disorder markedly worsens in single diaphragm muscle fibres within a 10-day course of MV+NMBA, modelling *Ventilator-induced Diaphragm Dysfunction* (VIDD) [33]. As the morphometry approach hitherto only covered an XY-image-wise analysis neglecting the myofibrillar vector order in z-direction, we recently improved and validated our *boundary tensor*-based algorithm in the same VIDD model to also include the z-information throughout an imaged fibre to reflect a true CAS3D [34].

In an attempt to trial novel treatment regimens for VIDD and CIM, small molecules, i.e., chaperone co-inducer BGP-15 [35,36] or dissociative glucocorticoid (DGC) Vamorolone, (17α,21-Dihydroxy-16α-methylpregna-1,4,9(11)-triene-3,20-dione, originally developed as VBP-15) [37,38] were applied to the experimental ICU rat model and showed a restoration of force-generating capacity, both in diaphragm and limb muscles. In a previous study, we were then interested in whether those small molecules were also capable in restoring or preventing the decline in myofibrillar architecture during the course of ICU intervention (MV+NMBA). This proved to be the case for both 2D [33] and 3D quantitative morphometry approaches [34], both being beneficial over the use of VBP-15’s mother compound Prednisolone, a classical anti-inflammatory steroid with yet more marked muscle catabolic effects [37].

As limb muscles are usually more severely affected by the complete mechanical silencing during the ICU condition (MV+NMBA), it would be important to know whether BGP-15 and/or VBP-15 are also effective here, and to what extent, compared to diaphragm. For this, no information is available to date. Thus, the present study was designed to test the hypothesis that small molecule BGP-15 orVBP-15 treatment was a potent global treatment regimen for both VIDD and CIM. We show that, using our 3D-quantitative morphometry approach, exploratory chaperone co-inducer or DGC treatments showed some tendency to delay ultrastructural disorder when both used in isolation; however, this tendency seemed to fall somewhat behind the more beneficial effects seen in VIDD.

## 2. Materials and Methods

### 2.1. Rat ICU Model and Pharmacological Interventions

**Figure 1:**
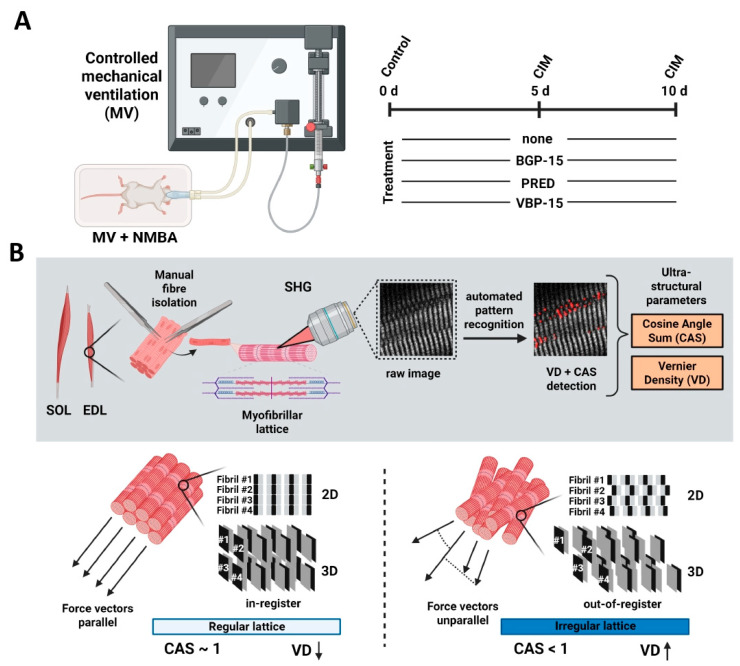
Experimental workflow of the rat ICU model, pharmacotherapy and single muscle fibre label-free multiphoton *Second Harmonic Generation* (SHG) morphometry. (**A**), mechanical ventilation (MV) and neuromuscular blockade (NMBA) of a rat over the course of 10 d. Rats were either subjected to no drug treatment (‘none’) or treated with either Prednisolone (PRED), BGP-15 or VBP-15. (**B**), single fibres were manually isolated from *soleus* (SOL) and EDL muscles harvested at day 0 (control), 5 or 10, and subjected to SHG imaging, followed by quantitative morphometry applying custom-made image processing algorithms. The sterical order of myofibrillar motor protein arrays is reflected by two parameters: ‘Cosine Angle Sum’ (CAS) and ‘Vernier Density’ (VD), reflecting angular disarray and axial out-of-register alignment of adjacent myofibrils, respectively. CAS was analysed with a slice-wise 2D CAS and a recently developed true 3D approach. The bottom schematics visualise typical patterns in healthy, i.e., regularly organised, sarcomeres and in an irregular lattice with angular and axial distortions.

To study the effects of small molecules (BGP-15 or VBP-15) or Prednisolone treatments on single limb skeletal muscle fibres’ sarcomere structure in 3D during ICU intervention, a rat ICU model [6,39] was employed. The group of animals included in this study consisted of 26 healthy adult female Sprague-Dawley rats, weighing 314 ± 6 g. Animals were randomly divided into one control group and four treatment groups. All groups were exposed to controlled mechanical ventilation (MV), neuromuscular blockade (NMBA) and deep sedation for five or ten days with either no drug treatment (ICU group ‘none’, *n* = 5), with Prednisolone (ICU group ‘PRED’, *n* = 5), with VBP-15 (ICU group ‘VBP-15’, *n* = 5), or BGP-15 (ICU group ‘BGP-15’, *n* = 6) (Figure 1A). The study’s control group was just exposed to MV and NMBA and immediately sacrificed (day 0), which was deemed a better control than completely untreated healthy animals. A brief description regarding sedation, anaesthesia, surgery, protein and fluid balance, parental solutions and monitoring of the animals can be found in our preceding study from Mnuskina, Bauer [33], and fully detailed descriptions can be found elsewhere [5,40,41]. In brief, Prednisolone 5 mg/kg/day (Prednisolone Alternova, Alternova, skælskør, Denmark), BGP-15 40 mg/kg/day (N-Gene Research Laboratories Inc., New York, NY, USA), and VBP-15 at 20 mg/kg/day (ReveraGen BioPharma, Rockville, MD, USA) were administered by oral gavage.

After the experimental period of five or ten days, depending on the respective experimental group, all animals were sacrificed, and the limb muscles of interest were manually dissected [36,37]. Limb muscles were from the same animal cohorts as in our previous study on diaphragm [33].

### 2.2. Chemically Skinned Skeletal Muscle Fibre Preparation

Once the muscles were dissected, all samples were prepared for subsequent cryo-storage according to the protocol given by Corpeno Kalamgi, Salah [39] and Renaud, Llano-Diez [42]. Briefly, the muscles were stored in cold relaxing solution (in mM: 4 Mg-ATP, 1 free Mg^2+^, 20 imidazole, 7 EGTA, 14.5 creatine phosphate; free Ca^2+^ 10^−9^ M; KCl to adjust ionic strength to 180 mM, pH adjusted to 7.0), subsequently tied to glass capillaries under slight stretch to remove any slack and then chemically skinned for 24 h at 4 °C, by adding 50:50 *v*/*v* glycerol. The skinned muscle samples were subsequently treated with increasing concentrations of sucrose (0.5–2 M) to protect them from cryo-damage. They were then removed from the capillaries, snap-frozen in nitrogen-chilled propane and stored at −160 °C to allow for long-term storage and sample rotation between the laboratories [43]. One day prior to experiments, the sucrose was gradually washed out of the samples by successively incubating them for 30 min in decreasing sucrose concentrations before storage in relaxing solution. For single fibre extraction, the muscles were stretched to their full length (i.e., removing slack), pinned down onto a polydimethylsiloxane-coated (PDMS, *Sylgard*, Dow-Corning, Wiesbaden, Germany) Petri dish and fixed by storing them in 4% paraformaldehyde in PBS at 4 °C overnight. After exchanging the solution for normal PBS, single fibre segments were manually tethered from the muscle using fine forceps (type 55, Dumont) under a laboratory stereomicroscope (Nikon SMZ800N, Nikon, Düsseldorf, Germany). Eventually, single fibres were transferred to a custom-made optical recording chamber and positioned for imaging by pushing both ends, under slight stretch to remove slack, into opposite streaks of petroleum jelly.

### 2.3. Second Harmonic Generation (SHG) Imaging

SHG imaging of single muscle fibres was realised using an ultra-fast laser-scanning multiphoton microscope (TriMScope II, LaVision BioTec, Bielefeld, Germany) coupled with a mode-locked femtosecond-pulse Ti:Sa laser (Chameleon Vision II, Coherent, Santa Clara, CA, United States) tuned to 810 nm, similar to the setup used in Mnuskina, Bauer [33]. On the excitation side, the laser was focused into the sample through an ×40 water immersion objective with a numerical aperture of 1.1 (LD C-Apochromat, 40×/1.1, Carl Zeiss, Jena, Germany). For myosin-II SHG detection in transmission mode, the detection side was equipped with another water immersion objective with a magnification of ×20 and a numerical aperture of 1.0 (W Plan-Apochromat, 20×/1.0, Carl Zeiss, Jena, Germany). From there, the SHG signal was sent through a 405/20 nm bandpass filter (CHROMA ET 405/20×, Chroma, Olching, Germany) and detected by an ultrasensitive transmission photomultiplier tube (H 7422–40 LV 5M, Hamamatsu Photonics, Herrsching, Germany). To ensure linear light polarisation and, therefore, optimal signal intensity [27], an adjustable, mechanically actuated half-wave (λ/2) plate was placed into the illumination light path, right at the back aperture of the excitation objective. The polarisation angle was individually adjusted for every sample to maximise for SHG signal intensity. The pixel size was set to 1024 × 1024 pixels per image with a physical image size of 200 × 200 µm, a line-scanning frequency of 600 Hz and a step size in axial direction of 1 µm. These parameters resulted in a pixel size of 0.195 × 0.195 × 1 µm at a pixel dwell time of 1.41 µs. From each sample, three individual XYZ volumetric image stacks were recorded, to be able to extract three-dimensional structural and morphological label-free SHG data. Imaging was performed by two co-authors to which the exact condition of the samples (being labelled MV+NMBA for controls, none/VBP/BGP for treatment groups, day of experiment termination, with animal identifier and muscle type) was unknown and can thus be considered blinded to the data acquisition.

### 2.4. Pattern Recognition Image Analysis and Quantitative Morphometry

The analysis included five different parameters: (i) Fibre Diameter (d); (ii) Sarcomere Length (SL); (iii) 2D-Cosine Angle Sum (CAS2D); (iv) 2D-Vernier Density (VD); and (v) 3D-Cosine Angle Sum (CAS3D). The fibre diameter was extracted in 2D by using the *Straight Line* tool in Fiji (https://fiji.sc/, accessed on 10 May 2026; licenced under the GNU General Public License) at three individual positions, to determine the largest diameter of every fibre. The two-dimensional CAS (CAS2D) and the VD were calculated using a self-developed algorithm described in Friedrich, Both [30] and Garbe, Buttgereit [32]. The CAS is a quantitative morphometry parameter that represents the amount of angular disarray between adjacent myofibrils within the analysed field of view within the XY-plane (CAS2D), and in a more recent improvement, also extending to 3D (CAS3D) [34]. CAS values close to unity represent a parallel alignment of adjacent myofibrils, while decreasing values indicate a higher disorder towards randomly oriented isotropic ultrastructure [28]. Verniers are Y-shaped patterns that occur due to axial in-register misalignments of otherwise Z-line registered myofibrils [33]. The VD in counts per 100 µm^2^ (#/100 μm^2^) is a parameter to quantify their abundance on a specified muscle cross-sectional area [30]. Vernier Densities close to zero indicate an undisrupted, perfectly alternating sarcomere pattern, while higher values indicate an increasing degree of Y-shaped (out-of-register) disruptions within that pattern. To add another layer of dimensional complexity and, therefore, to allow for even deeper insights into the morphological ultrastructure of muscle fibres, the two-dimensional CAS-algorithm was recently expanded and optimised to analyse image data in three dimensions simultaneously. Instead of averaging and adding up the angular information of every image slice to gain information about the muscle fibre as a whole (i.e., 2.5D approach), the new algorithm utilises Fourier space and real-space 3D analysis algorithms [34] to give direct insight into the volumetric 3D orientation of the myofibrils. A graphic representation of the different dimensional algorithmic approach is depicted in Figure 1B. In addition to the three-dimensional CAS-value (CAS3D), it also extracts the average sarcomere length of the fibre directly from the 3D image data. Of note, that image processing algorithm automatically performs on dedicated folder structures of the stored image data, therefore, eliminating human bias to data analysis The functional principle and validation of the newly developed algorithm can be found in Schneidereit, Bauer [34].

### 2.5. Statistical Analysis and Data Gaps

Statistical analysis was performed using Origin Version 2021 (OriginLab Corporation, Northampton, MA, USA) and was executed according to the analysis protocol given in our preceding study [33]. Once a whole XYZ stack was secured from a single fibre, all data were included in the analysis, and no fibre exclusion was applied. From each muscle of one animal, multiple single fibre recordings were obtained, in total yielding *n* independent biological replicate measurements from *m* animals. As such, the biological unit for statistics was considered to be the single fibre level, and statistical analysis followed a random effects model [44]. Briefly, a two-way analysis of variance (ANOVA) was applied to all datasets to screen for significant differences within the groups of variables ‘ICU duration’ (days 0, 5, 10) and ‘treatment’ (BGP-15, VBP-15, Prednisolone), as well as between the groups using a Tukey post hoc analysis. Afterwards, one-way ANOVA tests (followed by Tukey post hoc analysis) were applied to the data to extract further information, such as (i) the influence of MV+NMBA duration compared with the control group (ctrl), as prolonged ICU treatment (MV+NMBA) *per se* is expected to already affect the cytoarchitecture; (ii) the impact of any pharmaceutical treatment at a specific day compared with the untreated (‘none’) ICU-group, to assess whether any treatment shows an effect in preventing or ameliorating myofibrillar disorders during the course of the ICU condition; and (iii) the determination, whether any treatment shows the capability to restore morphology back to the state of a healthy animal (i.e., control group at day 0). The results were considered significant at *p* < 0.05 and highly significant at *p* < 0.001.

It has to be mentioned that due to the logistical challenges of transferring samples internationally, some sample containers were either damaged or opened at customs and, therefore, lost (unfixed biological material requiring to be inspected by a customs vet). In addition, during initial extraction from the cryopreserved state, some samples also suffered irreversible damage and could not be used for meaningful extraction of single fibre segments. Details on the sample distribution regarding successful and lost samples, alongside numbers of single fibres retrieved from muscles from the given number of animals and treatment groups, are listed in Table S1.

## 3. Results

### 3.1. SHG Imaging and Quantitative Morphometry Reliably Establish Sarcomere Architecture Dissolution with Marked Myofibrillar Disorder and Fibre Atrophy During the Course of ICU Intervention

**Figure 2:**
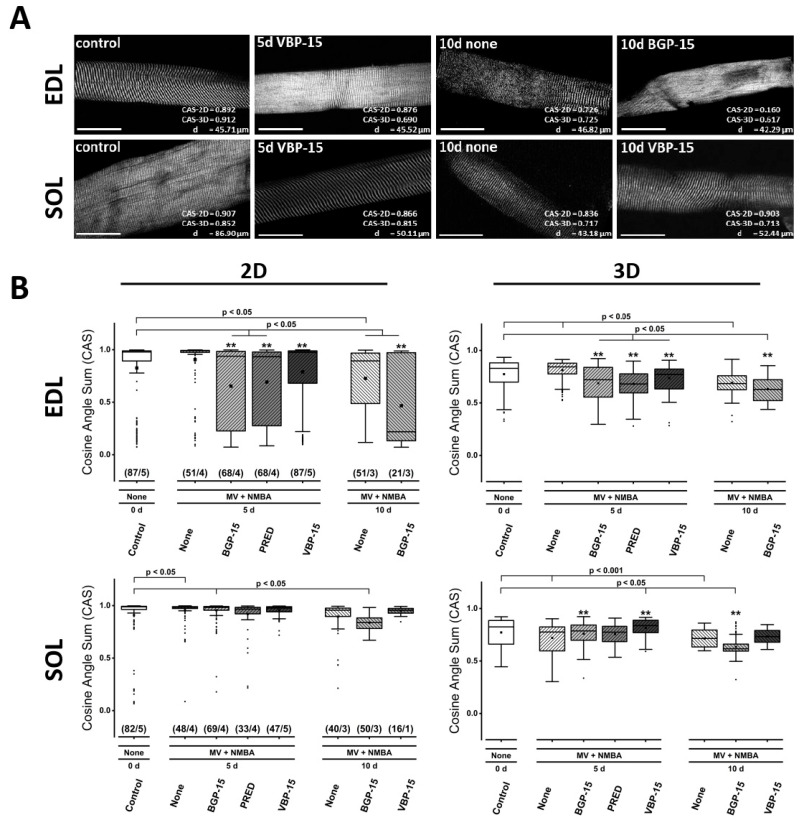
Myofibrillar lattice angular geometry of single *soleus* and EDL muscle fibres following 5 or 10 d MV+NMBA intervention without (none) or with PRED, VBP-15 or BGP-15 treatment. (**A**), middle plane images from within an XYZ SHG image stack are shown for single EDL (**top**) and *soleus* muscle fibres (**bottom**) at day 0 (control) and following 5 or 10 days of VBP-15 or BGP-15 treatment. As indicated for each image (each XYZ stack, respectively), the two-dimensional (CAS2D), the three-dimensional (CAS3D) *Cosine Angle Sum* (CAS), and the fibre diameter (d) are given. (**B**), statistical comparison of CAS values during the course of ICU intervention reveals a significant decline in untreated ICU rats (‘None’) in both muscles. Within each duration group (5 d and 10 d), neither treatment (BGP-15, VBP-15, PRED) could ameliorate the decline in CAS values (CAS2D and CAS3D) in EDL muscles but instead, further increased myofibrillar disorder (significantly smaller CAS values as indicated by asterisks). For *soleus* muscle, the different treatments did not negatively impact myofibrillar order (except for BGP-15 at day 10 in CAS3D). Instead, they significantly prevented the decline seen in the untreated rats. VBP-15 treatment seemed to preserve CAS-levels at those seen in the control (i.e., not being significantly decreased vs. 0 d). **: *p* < 0.05 vs. ‘None’ within the same time bin. Scale bar: 50 µm. (*n*/*m*): *n* fibres from *m* animals.

Figure 2A shows representative images from the middle plane of single *soleus* and EDL fibres at the beginning of the ICU intervention (MV+NMBA, control, day 0) and its ongoing course at 5 or 10 days for the indicated treatments. For the moment, the focus shall be on the ‘no treatment’ condition (‘none’) which impressively documents that at day 10, the regular sarcomere SHG pattern is severely compromised. The images clearly suggest a marked dissolution of the regular sarcomere lattice with wavy and grainy spotted areas within the fibre. This process seems to be much more prominent in fast-twitch EDL fibres than in *soleus* (SOL) fibres. This is also confirmed by the morphometry analysis extracting the *Cosine Angle Sum* (CAS) of myofibrillar angular orientations, both in 2D as well as in our recently improved true 3D analysis, taking into account orientation information in the z-direction (Figure 2B). During the course of ICU intervention without drug treatment, both muscle types were affected and showed significant reductions in CAS values. CAS3D analysis seemed more sensitive in picking up decline in myofibrillar order already after 5 days of ICU condition which was less consistent for CAS2D. Once significantly declined at day 5 in the CAS3D analysis, a persistent further decline was statistically assessed with CAS3D, but not with CAS2D (i.e., CAS2D in SOL muscle being significantly declined after 5 days but no longer so after 10 days). Also, the analysed data seemed to be more compact with CAS3D, either showing smaller variations (i.e., in EDL) or fewer outliers (e.g., in SOL) compared to CAS2D. While CAS2D analysis suggests a much more severe affection of myofibrillar disorder in EDL over SOL muscle fibres (however, with much larger data variability), CAS3D, taking into account all spatial axes contributions to angular orientations, suggests a more similar affection in both muscle types. Nevertheless, both analyses reliably map the marked dissolution of myofibrillar order during the course of critical illness.

**Figure 3:**
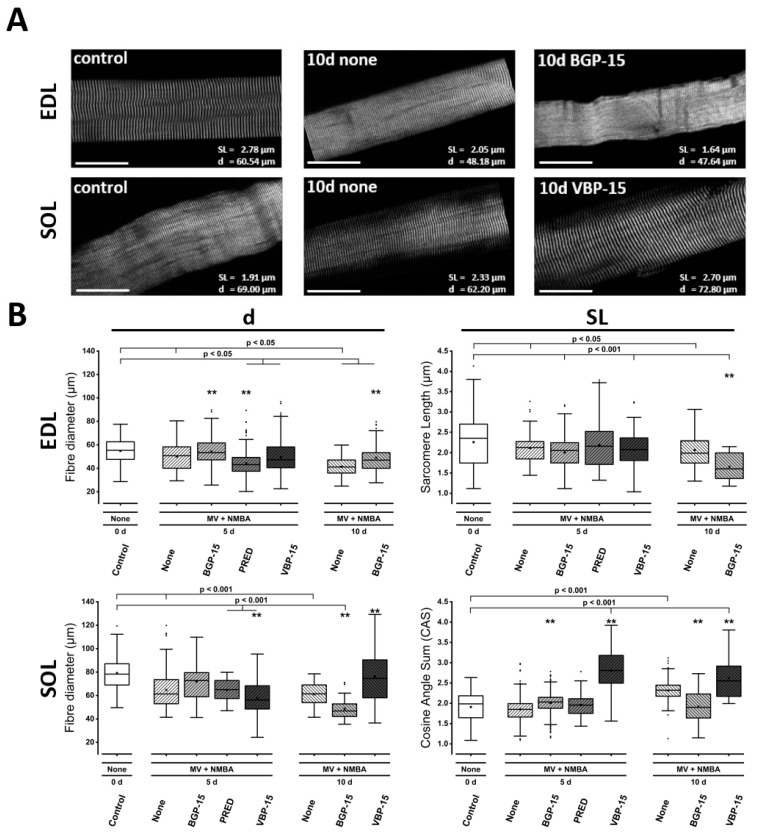
Diameter (d) and resting sarcomere length (SL) of single *soleus* and EDL muscle fibres following a 5 or 10 d MV+NMBA intervention without (none) or with PRED, VBP-15 or BGP-15 treatment. (**A**), middle plane images are shown for single EDL (**top**) and *soleus* muscle fibres (**bottom**) at day 0 (control) and following 5 or 10 days of applying VBP-15, BGP-15, or no treatment (‘none’). Fibre diameter and SL values are given for each fibre. (**B**), statistical comparison of diameters (**left**) and SL values (**right**) for EDL (**top**) and SOL (**bottom**) muscle fibres over the course of ICU condition and treatment regimens. A significant decline in fibre diameters can be seen in both muscles, worsening with duration. BGP-15 slowed down atrophy compared with no treatment in EDL fibres but was less efficient in SOL fibres, leading to even more pronounced atrophy. In contrast, VBP-15 ameliorated atrophy in those fibres after 10 days of treatment. As for sarcomere lengths, those were significantly declined already after 5 days of ICU condition in EDL fibres while not being altered in SOL fibres at day 5 but significantly increased at day 10. BGP-15 had no major effects at day 5 in EDL fibres, but induced a significant decrease at day 10. BGP-15 effects in SOL fibres were inconsistent while VBP-15 resulted in a robust increase in SL over time that even surpassed the SL values seen in control fibres. **: *p* < 0.05 vs. ‘None’ within the same time point of ICU intervention. Scale bar: 50 µm.

Figure 3 depicts the analysis of fibre diameters (d) and resting sarcomere lengths (SLs) for the same conditions as above. Again, the focus shall be on the ‘none’ case, which clearly shows progressing fibre atrophy with ongoing ICU condition, both in representative XY images (Figure 3A) and the statistical analyses (Figure 3B) in both muscle types. Fibre diameters were significantly reduced following both 5 and 10 days of ICU condition, which was significant for EDL and highly significant for SOL fibres. Sarcomere lengths also showed a progressing, significant decline during critical illness, with an earlier onset for EDL (already significantly declined after day 5) as compared with SOL fibres which were not changed at day 5 but instead, highly significantly increased at day 10.

**Figure 4:**
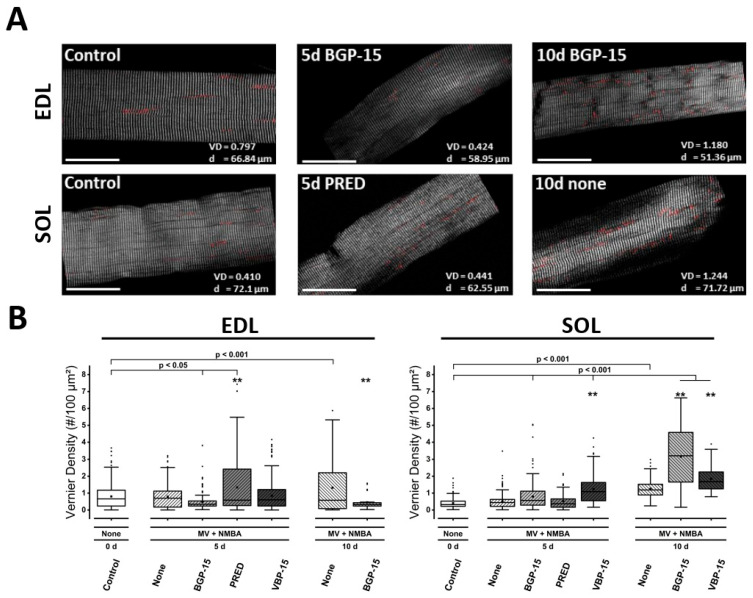
Vernier Densities (VD) in single *soleus* and EDL muscle fibres following a 5 or 10 d MV+NMBA intervention without (none) or with PRED, VBP-15 or BGP-15 treatment. (**A**), middle plane images are shown for single EDL (**top**) and *soleus* muscle fibres (**bottom**) at day 0 (control) and following 5 or 10 days of applying PRED, BGP-15 or no treatment (none). VD values and fibre diameters are given for each fibre. (**B**), statistical comparison of VD values in EDL (**left**) and SOL (**right**) muscles over the course of ICU condition and for the given treatment regimens. During the course of ICU condition, a significant increase in myofibrillar lattice misalignment (i.e., increased VD values) was seen in both muscle types, becoming significant at day 10. Within each ICU duration group, BGP-15 was able to markedly reduce VD values in EDL muscle, while acting oppositely in SOL muscle fibres. This effect was also seen by VBP-15 in SOL muscle (note: for EDL muscle, no VBP-15 samples were available). BGP-15 restored VD levels back to control fibre levels in EDL muscle at day 10. In SOL muscle, no small-molecule treatment was capable of restoring VD levels back to control levels. **: *p* < 0.05 vs. ‘None’ within the same time point of ICU intervention. Scale bar: 50 µm.

As a second measure for myofibrillar disorder, the axial lattice shifts, seen as *Vernier Densities* (VD) in the regular striation pattern [30], were analysed and are shown in Figure 4. Under control conditions (0 days), VDs in both muscle fibre types were below 1/100 µm^2^, consistent with previous observations [28,29]. In both muscle types, VD values increased during the course of ICU condition, which was not yet significant at day 5 but highly significant at day 10, indicative of substantial axial lattice disruption in both fast- and slow-twitch muscle.

Lastly, in an attempt to test the hypothesis that the structural irregularities seen in the myofibrillar scaffold during the course of the ICU intervention was linked to fibre atrophy, we performed Pearson’s correlations of the respective SHG morphometry parameters CAS3D and VD against fibre diameters d. Figure S1 shows the linear correlations to the individual data clouds for 0 days (black), 5 days (red) and 10 days (green) of MV+NMBA. For both *soleus* and EDL fibres, a shift towards smaller diameters is discernible with critical illness duration. Pearson correlation coefficients were assessed for statistical significance and are tabulated in Table S2. Seven out of the 12 tested correlation pairs turned out to be non-significant, and even in cases where significances demonstrated a trend with ICU duration, those were mostly inconsistent (e.g., compare r values in *soleus* fibres being negative for diameter and VD at day 5 but positive at day 10, or significances at day 0 disappearing for later time points). Of note, at day 10, where fibre atrophy was most prominent, only one out of four correlations’ *p*-values was significant (EDL, 10 days, diameter vs. VD).

### 3.2. Effects of Small- Molecule Treatment with BGP-15 and VBP-15 Compared to Prednisolone on the Myofibrillar Architecture in Soleus and EDL Muscle Fibres During ICU Intervention

Similar to our previous study on diaphragm muscle, we analysed the effects of treatments with either small molecule VBP-15 or BGP-15 against Prednisolone on the ultrastructural order parameters CAS2D/3D, VD, d and SL in *soleus* and EDL fibres. As for the *Cosine Angle Sums*, both for CAS2D and CAS3D analyses, none of the mentioned treatments was capable of preventing the CIM phenotype in EDL muscle (Figure 2B). Instead, all three treatment regimens seemed to significantly worsen angular orientation alignment, as indicated by the asterisks comparing the treatment outcome to the no treatment (‘none’) condition for that same day. While myofibrillar disorder was even further promoted by small molecules or Prednisolone treatment in EDL, in *soleus* muscle, the treatments did not negatively impact myofibrillar order (except for BGP-15 at day 10 in CAS3D). Instead, they significantly prevented the decline seen in the respective untreated MV+NMBA rats (‘none’). VBP-15 treatment seemed to robustly preserve CAS levels at those seen in the control (i.e., not being significantly decreased vs. 0 d) while no other treatment could restore control levels.

Regarding fibre atrophy during the course of ICU condition, BGP-15 was able to potently slow down the decline in fibre diameter as seen by the still significantly larger d values as compared to the ‘none’ group at each time point and being similar to controls at day 5; however, diameters were significantly declined compared with controls at day 10 (Figure 3B, left panels). In SOL fibres, this effect was not that pronounced, with d values still comparable to control values at day 5, but then being significantly declined both compared with control values and the ‘none’ values at day 10. Instead, while VBP-15 could not prevent decline in diameters at day 5 and at day 10, diameters were significantly larger than the ICU group (‘none’) and even restored to control values. Unfortunately, for EDL fibres, no data were available for VBP-15 at day 10 due to very bad conditions of samples preventing single fibre isolation or loss of samples during sample transfer.

As for sarcomere lengths, neither of the treatments showed major effects at day 5 in EDL fibres; however, a significant decrease occurred at day 10. For SOL fibres, BGP-15 effects were inconsistent while VBP-15 induced significantly larger SL values that even highly significantly surpassed the values seen in control fibres (Figure 3B, right panels).

The axial lattice order, as judged from the VD values, was robustly preserved by BGP-15 in EDL fibres as seen by VD values lowered even below control values, while both Prednisolone and VBP-15 increased VD values compared to controls and the untreated ‘none’ group (significant for Prednisolone). For the SOL samples where data on VBP-15 treatment were available throughout, neither BGP-15 nor VBP-15 were able to prevent axial lattice disruptions, with VD values rising highly significantly compared with controls during ongoing ICU condition, even significantly above the ‘none’ groups for VBP-15 at day 5, and both VBP-15 and BGP-15 at day 10, indicating an adverse effect on axial lattice order in slow-twitch fibres.

## 4. Discussion

Remodelling of tissue and cellular structure is an important process during health, ageing and acute and chronic disease states. It fuels the structure–function dogma, i.e., the structural imprint on function on longer time scales. This is particularly true for skeletal muscle where myofibrillar remodelling with changes to the sterical arrangement of normally perfectly aligned and registered sarcomeres directly correlates with the predicted force-generating capacity [45]. So far, a detailed morphometry approach of ultrastructural disorder over the course of the ICU condition, i.e., MV+NMBA, has been missing. On a level more accessible to low-resolution widefield imaging, in an early study in patients with ‘acute quadriplegic myopathy’ (AQM, an early descriptive term for critical illness myopathy), single *tibialis ant.* muscle fibres showed the hallmarks of CIM, i.e., marked single fibre atrophy, a decline in specific isometric tension and electrophoretically documented loss of myosin [4]. This was also confirmed in a recent study monitoring single *tibialis ant.* muscle fibre gross structure and function in ten neuro-ICU patients exposed to long-term MV, where massive fibre atrophy of ~30% of initial values and even larger declines (~50%) of CSA-normalised specific force were found in repeated biopsies being taken over 12 days [12]. It is clear from the CSA normalisation that the force loss cannot be explained by atrophy (which would be expected to maintain specific force, if atrophy was the sole cause of single fibre weakness). Moreover, besides activation of protein degradation pathways and downregulation of myofibrillar protein synthesis [12], an altered sterical ultrastructure could also have accounted to a marked extent to the observed degenerate ‘quality of force’. However, although that study could not exploit the means of specific, label-free multiphoton imaging of myosin-II SHG, the brightfield images shown in their Figure 1 already point towards some axial lattice shifts being adumbrated [12].

### 4.1. Myosin Loss and Increasing Myofibrillar Disorder in EDL and Soleus Single Fibres During a 10-Day Course of ICU Intervention in the Rat

As electrophoretic analysis of myosin content from single fibres can only assess the global protein content without any spatial information, we previously used SHG imaging in single *soleus* muscle fibres from the rat ICU model in a preliminary study to answer the question of whether the signal loss inherent to myosin-II was evenly distributed within affected fibres or whether it was more like a pattern associated with ‘hot spots’ within the fibre as a nucleus for proteolysis [46]. That initial study already demonstrated that SHG signal intensities were significantly reduced after 5 days of ICU intervention, indicating a homogeneous loss of myosin across the entire fibre, thus refuting the ‘hot spot’ hypothesis. Also, fibre atrophy was already severe, with approximately 25–30% reduction in fibre diameter. Interestingly, neither Vernier Densities (VD), nor CAS (i.e., CAS2D) were significantly altered at that time point [46].

In the present study involving higher animal numbers than previously, the similarity between VD at day 0 and day 5 in SOL fibres was confirmed, but it became significantly different at day 10 (Figure 4B). This suggests that axial lattice disruptions take longer to develop than, e.g., the angular myofibril deviation as seen in the CAS2D and CAS3D values which were now significantly reduced here in *soleus* fibres also already on day 5, but much more clearly so at day 10. The marked atrophy seen in our previous work [46] is also reflected here from day 5 onwards and is in line with an about 40% reduction in *soleus* fibre cross-sectional area seen following eight days of MV and immobilisation in the rat [47]. Regarding fast-twitch EDL muscle, the time course and extent of atrophy mirrored that of slow-twitch SOL single fibres from day 5 to 10 (Figure 3B). The same applied to the degree of myofibrillar order which, like in *soleus*, was unaltered for axial lattice register (VD) in EDL at day 5 but significantly out-of-register at day 10. The angular disorder was also similar. The two-dimensional CAS2D showed some inconsistency between *soleus* and EDL fibres regarding the myofibrillar disorder at both time points. However, when judged from the more relevant three-dimensional CAS3D that also takes into account deviations in myofibril vectors in z-direction [34], both muscle types showed a similarly progressing deviation from the parallel filament alignments over the time course of the ICU condition. This is in line with previous findings in the same rat ICU model showing that single fibres from the unloaded (mechanically silenced) limb side showed significantly smaller CSA values after an eight-day ICU intervention, in both *soleus* and EDL fibres, with a larger atrophy in the EDL [48]. Yet, another study showed significantly more atrophy in *soleus* fibres and still comparable fibre CSA values in EDL compared with controls for a 5-day ICU intervention, albeit, both with significantly (~15%) reduced specific force capacity [37].

Interestingly, a passive loading regimen was able to ameliorate both CSA values and specific force in the loaded limb side muscle fibres, with a restoration almost to the level of controls (also 0 day MV+NMBA) in the *soleus* but not in the EDL [48]. Since in the present study, no loading was involved, the effects of ICU intervention on the fibre diameters are comparable. The generally marked atrophy seen in both limb muscles (*soleus* and EDL) in CIM during ongoing ICU intervention is a direct consequence of the combination of MV and mechanical silencing [20,48]. In contrast, rodent models of LPS-induced peritoneal sepsis with animals still mobile and conscious showed marked (~30%) CSA atrophy in the fast-twitch EDL but almost no change in the load-bearing slow-twitch *soleus* 24 hr post LPS [49]. Overall, type II fibres have been found to be affected more than type I fibres in sepsis-induced muscle weakness, while disuse of muscles more prominently affects type I fibres [50]. Therefore, since the ICU condition involves both immobilisation as well as inflammatory cytokine releases during MV [12], myopathic changes are expected to be seen in both fast- and slow-twitch fibres.

Although atrophy is undoubtedly one of the major factors for the decline in absolute force seen in CIM, MV+NMBA produce much more detrimental effects to the quality of contraction. This has been seen in the marked decline in specific force, both for EDL and *soleus* single fibres [37]. Apart from the involvement of many anabolic and catabolic pathways or Ca^2+^ signalling [21,47,51], a still unanswered contribution may be attributed to myofibrillar remodelling as reflected by our current results. It is well established that angular deviations in myofibril alignment from the normal parallel configuration alongside disruptions of the lattice register represent a structural component of compromised force generation [29,30] that is expected to contribute to the decline in specific force. This is because the internal force-generating hierarchy is not reflected in the pure measurements of fibre diameters or CSA values. A direct correlation of isometric force capacity to SHG-derived morphometry values (VD, CAS2D) suggested a linear relationship between force and myofibrillar order [45]. Using a rough estimation using our 10-day MV+NMBA CAS2D median values (~0.89) versus control EDL fibres (~0.97) in the direct force-CAS(2D) relationship for single EDL fibres presented in Schneidereit, Nübler [45], this would correspond to a projected force drop of approximately 30% during the course of the ICU condition. Although this can only be a first indirect approximation for force prediction from CAS values in the EDL fibres using a calibration established from a different remodelling pathology (*Duchenne* muscular Dystrophy in Schneidereit, Nübler [45]), those fall within the range of about 20% decline in median single EDL fibre specific force seen in the rat ICU model following 5 days [37]. A direct correlation of specific force-CAS2D/3D data pairs from single fibres would be required over the course of ICU intervention using our *MechaMorph* technology in future studies to experimentally verify this estimate.

Interestingly, we could not detect any consistent correlation between fibre atrophy and SHG morphometry parameters CAS3D and VD during ongoing ICU intervention (Figure S1 and Table S2), suggesting that myofibrillar disorder represents an additional structural feature of CIM rather than being atrophy-related.

### 4.2. Effects of BGP-15 and VBP-15 Small-Molecule Treatment on Single Fibre Myofibrillar Architecture During a 10-Day Course of ICU Intervention in the Rat

Previous studies have shown marked alterations in muscle protein expression under the stress conditions of MV and complete mechanical silencing. For instance, in a pig ICU model, MV+NMBA was found to deteriorate heat shock protein 70 (HSP-70) expression, a chaperone expressed under oxidative or heat stress to maintain normal protein folding balance [21]. Hsp72 was found to be reduced in adult and aged rats during muscle disuse protocols [52]. In the pig ICU model, HSP-70 expression was several-fold upregulated in the *masseter* muscle over the *biceps brachii* after 5 days of ICU intervention, indicating that an increase in chaperone expression could be a major factor to explain the sparing of facial *masseter* muscle from muscle weakness in CIM over limb muscles [53]. In an attempt to artificially increase muscle chaperone expression, administration of the HSP-72 co-inducer BGP-15 in the rat ICU model over a course of 10 days was found to improve *soleus* muscle single fibre specific force production in the early phase at day 5 but no longer for longer durations when the preferential myosin loss was already prevalent [54]. While in diaphragm muscle, unlike in the *soleus*, myosin:actin protein ratios were maintained over the 10 d course of ICU intervention; BGP-15 was also potent to restore the significantly declined specific force there [54].

VBP-15 belongs to a new group of ‘dissociative glucocorticoid’ (DGC) small molecules that is derived from Prednisolone, effectively dissociating the activation of anti-inflammatory and protein degradation pathways seen in other glucocorticoids [38]. Applying this to the rat ICU model, Akkad, et al. [37] found marked protection against fibre atrophy and specific force decline in *soleus* muscle that was even more pronounced in EDL muscle in rats treated with VBP-15 during a 5-day course of ICU-intervention. Also, animals showed a significantly improved survival rate. Prednisolone treatment, in contrast, markedly exacerbated the effects of ICU treatment, in particular in EDL muscle [37]. As the significantly reduced *soleus* and EDL myosin:actin ratios in the ICU group were maintained at control levels by VBP-15 with some underlying decreases in both actin and myosin heavy chain expression, the question of whether the maintained single fibre function derives, at least in part, from a preserved myofibrillar architecture, could not be answered to date.

In our current setting, we use the CAS2D/3D and VD as indicators of myofibrillar order. We have previously shown that such morphometry parameters significantly correlate with single fibre force output and are, thus, predictors of structure–function relationships [45]. In expansion to our previous CAS2D algorithms, the CAS3D provides a more holistic assessment of the three-dimensional angular arrangement of myofibrils over the CAS2D as it also interpolates the angles of sarcomeres extending into the z-plane, which was not possible before [34]. As a result, CAS3D values were shown to be more robust and lower in variability [34]. This can, for instance, be well seen for EDL fibres where CAS3D values were much more compact compared with CAS2D values, while the apparently larger scatter in the *soleus* fibres in CAS3D values (Figure 2B) probably reflects a true effect of more chaotic angle distributions into the z-plane that was not picked up by the more limited XY CAS2D. The CAS3D showed significantly better order for *soleus* fibres at day 5 with both BGP-15 and VBP-15 over the untreated ICU condition and was similar to control values. At day 10, however, BGP-15 did not provide any protective effect against myofibrillar remodelling as seen from significantly reduced CAS3D and increased VD values against the ICU condition *per se*. VBP-15 also did not ameliorate structure but also did not worsen it compared to the untreated condition. Those results show that it is important to perform ongoing validation of the recently introduced CAS3D methodology. It also justifies the side-by-side comparative CAS2D vs. CAS3D analyses in this study rather than only focusing on the CAS3D method.

We used the statistical tests for non-significance against the 0-day ICU control to determine whether sarcomere architecture could be preserved/restored close to control values. For 10 days of treatment, this was not the case for any treatment in either EDL or *soleus*. Instead, it seems that BGP-15 may have had some beneficial effect up to day 5 but not thereafter. This is in agreement with the single fibre specific force data in *soleus* muscle in Cacciani, Salah [54], showing partial protection until day 5 but even further deterioration at day 8 and day 10 in the same model. Judging from the architectural data, the CAS3D seems to have a higher predictive effect than the VD values in the overall order assessment. However, as mentioned above, an explicit structure–force assessment in the same fibre to predict force from structure as in [45] is still missing for this ICU model. For *soleus*, although specific force measurements did show some protection by VBP-15 from the ICU-induced weakness at day 5 [37], only the cytoarchitectural parameter CAS3D was in line with this: values were somewhat higher than controls at day 5 and similar to controls at day 10. However, VD values had the opposite effect towards more disorder, with values significantly rising at both day 5 and 10 above controls. This may be another hint towards myofibrillar angular distribution being more important for concerted force output than axial lattice registration. Of note, atrophy was markedly reversed for *soleus* fibres at day 10, but not for EDL fibres. As Akkad et al. [37] did only report on 5-day effects, it remains open whether VBP-15 has some effects that require longer times to develop their full potential. In contrast, BGP-15 seemed to preserve structure better in the short-term (5 days) but became detrimental at longer application times (10 days; see limitations below).

As the classical glucocorticoid Prednisolone, VBP-15’s mother compound with good anti-inflammatory, yet muscle catabolic, effects, is still being used to control systemic inflammatory response syndrome (SIRS) and other inflammatory ICU conditions [55], we also analysed available samples from Prednisolone-treated animals. Similar to our previous findings in diaphragm fibres [33], Prednisolone’s catabolic action was seen reflected in marked single fibre atrophy and myofibrillar disorder (high VD, low CAS2D73D values), more so in the EDL than in the *soleus*.

### 4.3. Comparison of Limb Muscle (Soleus, EDL − CIM) with Diaphragm (VIDD) in the Rat ICU Model

As we have previously conducted a similar morphometric label-free analysis of myofibrillar cytoarchitecture of diaphragm single fibres in conjunction with BGP-15 or VBP-15 treatment in the rat ICU model, the major findings for limb muscles for CIM in this study shall be briefly put into context with VIDD [33]. Diaphragm single fibres were more resistant to atrophy during the MV+NMBA intervention, with diameters only being significantly reduced at day 10 but not day 5. VBP-15, but not BGP-15, potently restored fibre diameters back to control levels at day 10. In this regard, limb muscles (both EDL and *soleus*) are somewhat more susceptible to ICU treatment-induced fibre atrophy as evidenced here and in Salah, Li [36]. Other studies have even reported a relative fibre hypertrophy in diaphragm during the 5-day course of ICU intervention in the rat [35]. Although not reversing diaphragm fibre atrophy, muscle fibre function was greatly improved at day 5 of BGP-15 treatment [54]. However, this does not seem to hold for limb muscles, as seen by no restoration effect in *soleus* specific force [54] or myofibrillar order (this study). Unlike the effects of BGP-15 on diaphragm force at day 5 in the aforementioned study, our previous SHG morphometry analysis showed a marked decline in myofibrillar order by day 5 with some restoration capacity for BGP-15 at day 10 as seen in the CAS2D [33]. The latter was also confirmed in our follow-up CAS3D analyses [34]. As mentioned above, VBP-15 had some marked restoration effect on single fibre diameter and VD but not on CAS2D/3D in the diaphragm at day 10 [33,34], while having a similar restoration effect on *soleus* fibre atrophy and CAS3D but not VD (this study). For EDL muscle, unfortunately, not all data sets were complete, and VBP-15 treated fibres at day 10 could not be assessed.

### 4.4. Conclusions, Limitations and Future Research

From the structural evidence in this and our previous study in diaphragm [33] as well as functional data from literature, it becomes apparent that the combination of MV and complete mechanical silencing via NMBA has a more detrimental effect to limb muscle in CIM as compared to diaphragm in VIDD. This is reflected by earlier onset of atrophy and preferential myosin loss as well as decline in specific force that is more pronounced in anti-gravitational slow-twitch *soleus* over fast-twitch EDL muscle, while both muscle types present a similar decline in myofibrillar disorder during a 10-day ICU intervention. Chaperone co-inducers, like BGP-15, as well as dissociative glucocorticoids, like VBP-15, may have some beneficial effects, although their temporal profile seems to be different, and functional force seems to benefit more than structural myofibrillar order. It is tempting to speculate that BGP-15 might be more suitable as an intervention in the early phase of critical illness (i.e., first five days) while VBP-15 may take longer to exert its beneficial effects (i.e., 5–10 days). For EDL muscle, no intact VBP-15 treatment samples were available. Therefore, more-powered studies with more complete sample bins are required to substantiate our current, rather exploratory drug study.

Apart from those conclusions, our study is prone to several limitations: a major limitation is the lack of co-treatment regimen, e.g., an early treatment with BGP-15 up to day 5, followed by ongoing application of VBP-15, or other combinations. This should be an important direction for future studies. Also, since human samples are becoming more available through prospective clinical trials in ICU patients [54], future work should also be dedicated to quantitative morphometry of myofibrillar sarcomere order in the human setting. Moreover, in order to bring structure and function together, a direct assessment of both in the same sample using, e.g., our *MechaMorph* technology [45], shall be a valuable option to perform a direct ‘force-from structure’ calibration. Lastly, generalisation of our findings has to be made with caution as the study design may be affected by pseudo-replication in treating multiple fibres from the same animal as independent observation and, due to the elaborate ICU rat model with small sample sizes, focusing on female gender only.

## Data Availability

Imaging data sets are available from the corresponding author upon reasonable request.

## Supplementary Materials

The following supporting information can be downloaded at: https://www.mdpi.com/article/10.3390/cells15131219/s1, Figure S1: Pearson correlation analysis of SHG morphometry parameters CAS3D and VD with fibre diameter in EDL and *soleus* muscle for the control group (0 d, black symbols), 5 d (red) and 10 d (green) of ICU intervention without treatment (‘none’); Table S1: Summary table of all experimental animals and the respective extracted fibres, classified according to time period, treatment method and muscle type; Table S2: Quantitative results to the Pearson correlation analysis.
