# Supplementary material for: ‘Weak by Structure’—Limb Muscle Fibre Cytoarchitecture Remodelling During Critical Illness and Effects of Chaperone Co-Inducer BGP-15 and Dissociative Glucocorticoid VBP-15
Source: Cells. 2026 Jul 4;15(13):1219. doi: 10.3390/cells15131219 (PMC13360227; doi:10.3390/cells15131219)
Supplement: Supplementary file 1 [file cells-15-01219-s001.zip › cells-4340293-supplementary.pdf]

Supplementary material

'*Weak by structure*' - limb muscle fibre cytoarchitecture remodelling during the course of critical illness and effects of chaperone co-inducer BGP-15 and dissociative glucocorticoid VBP-15

Julian Bauer[1], Sofia Mnuskina[1], Anette Wirth-Hücking[1], Michael Haug[1], Dominik Schneidereit[1], Sefanie Nübler[1], Lucas Kreiss[1], Sebastian Schürmann[1], Nicola Cacciani[2], Lars Larsson[2], Oliver Friedrich[1]

[1] Institute of Medical Biotechnology, Department of Chemical and Biological Engineering, Friedrich-Alexander University Erlangen-Nürnberg, Paul-Gordan-Str. 3, 91052 Erlangen, Germany

[2] Department of Clinical Sciences, Swedish University of Agricultural Sciences, Uppsala, SE-750 07, Uppsala, Sweden

Correspondence: oliver.friedrich@fau.de, lars.larsson@slu.se

Supplementary Table S1: Summary table of all experimental animals and the respective extracted fibres, classified according to time period, treatment method and muscle type.

Time	Animal	Treatment	Fibres EDL	Fibres SOL	Comments
0d	070220-2	None (Control)	18	16	
	80110		17	16	
	KI20		18	18	
	KI21		14	17	
	KI23		20	15	
5d	UU40	None	0	0	The samples were damaged during transport, and only the diaphragm of the animal remained intact.
	KI17		17	17	
	KI30		17	17	
	KI31		17	14	
	KI5	BGP-15	17	18	
	KI26		17	16	
	KI28		17	17	
	UU73		17	18	
	KI4	PRED	17	0	The SOL was damaged during the initial extraction and, therefore, not available for experiments.
	KI11		17	0	The SOL was damaged during the initial extraction and, therefore, not available for experiments.
	KI12		17	15	
	KI15		17	18	
	KI7	VBP-15	17	0	The SOL was damaged during the initial extraction and, therefore, not available for experiments.
	KI8		17	0	The SOL was damaged during the initial extraction and, therefore, not available for experiments.
	KI16		18	17	
	KI47		17	14	
	KI48		18	16	
10d	UU33	None	17	10	
	UU36		17	17	
	UU66		17	13	
	UU42	BGP-15	21	17	
	UU84-A		0	16	The EDL was damaged during the initial extraction and, therefore, not available for experiments.
	UU-84-B		0	17	The EDL was damaged during the initial extraction and, therefore, not available for experiments.
	KI18	VBP-15	0	16	The EDL was damaged during the initial extraction and, therefore, not available for experiments.

Supplementary Table S2: Quantitative results to the Pearson correlation analysis shown in Suppl. Fig. 1. *: p<0.05, **: p < 0.001.

Time	Muscle	Correlation	r-value	p-value	Comments
0d	EDL	Fibre diameter / CAS-3D	-0.144	0.022	*
		Fibre diameter / VD	-0.008	0.901	
	SOL	Fibre diameter / CAS-3D	0.056	0.500	
		Fibre diameter / VD	-0.259	0.002	*
5d	EDL	Fibre diameter / CAS-3D	0.136	0.095	
		Fibre diameter / VD	-0.154	0.059	
	SOL	Fibre diameter / CAS-3D	-0.442	<0.001	**
		Fibre diameter / VD	0.312	0.003	*
10d	EDL	Fibre diameter / CAS-3D	0.006	0.940	
		Fibre diameter / VD	-0.164	0.045	*
	SOL	Fibre diameter / CAS-3D	0.163	0.235	
		Fibre diameter / VD	0.197	0.148	

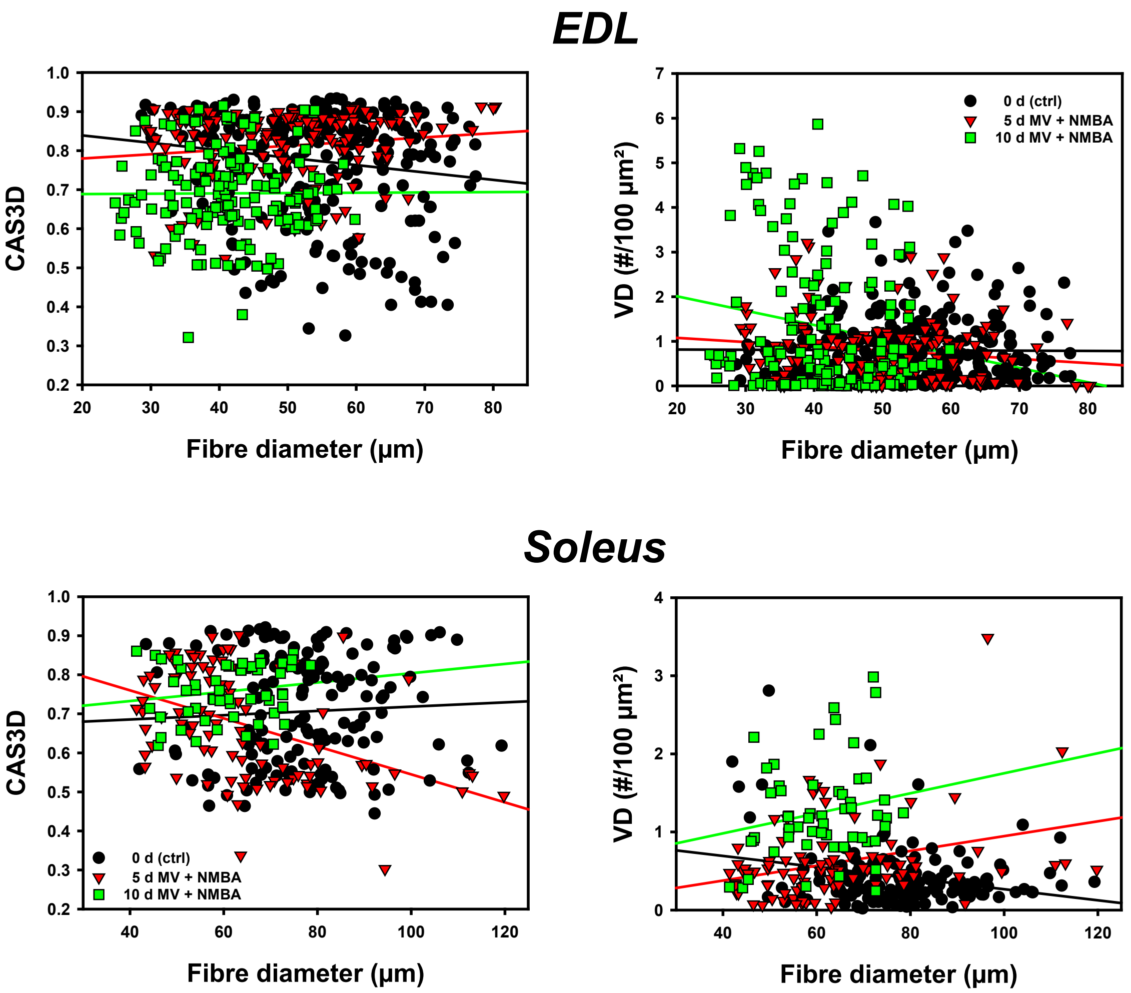

Supplementary Figure S1: Pearson correlation analysis of SHG morphometry parameters CAS3D and VD with fibre diameter in EDL and *soleus* muscle for the control group (0 d, black symbols), 5 d (red) and 10 d (green) of ICU intervention without treatment ('none'). The analyses were carried out to test for significant correlations between morphometry parameters and fibre atrophy (i.e., reduced diameter) during the course of MV + NMBA. The diameter distributions over the course of ICU interventions confirm a shift towards smaller fibre diameters, indicative of single fibre atrophy, both in *soleus* and EDL. However, as detailed in **Suppl. Table 2**, there were no consistent significances over time to warrant a dependence of myofibrillar disorder with ongoing atrophy.
